# Exploring the Skills Prioritised in the Assessment of Students Pursuing Adult Nursing, Post‐COVID: A Qualitative Analysis

**DOI:** 10.1002/nop2.70236

**Published:** 2025-04-30

**Authors:** Carmel Bond, Joanna M. Painter, Donna Barnett

**Affiliations:** ^1^ Department of Nursing and Midwifery, College of Health Wellbeing and Life Sciences Sheffield Hallam University Sheffield UK; ^2^ Rotherham Doncaster and South Humber NHS Trust Doncaster UK

**Keywords:** communication, compassion, content analysis, practice assessment, qualitative, student nurses, values‐based care

## Abstract

**Aim:**

To explore comments made by registered nurse practice assessors during the clinical assessment of students pursuing adult nursing.

**Background:**

Student nurses are entering a workforce that has changed professional and educational nursing practice systems due to the COVID‐19 pandemic. Limited placement learning during lockdowns reduced opportunities for students to practise clinical skills in person. However, the current state of practice assessment for students pursuing adult nursing, particularly post‐COVID, has yet to be explored.

**Design:**

Qualitative content analysis.

**Methods:**

Data were collected from the online assessment documentation of second‐year BSc students pursuing adult nursing. A qualitative content analysis was performed to analyse comments made by qualified adult nurses who are positioned as clinical practice assessors for students pursuing adult nursing. COREQ reporting guidelines were followed.

**Results:**

Two main themes were identified: (1) task‐focussed competencies and (2) relational aspects of care. Task‐focussed competencies included ‘technically complex physical health skills’ with an emphasis on specialist clinical interventions and procedures. Relational aspects of care included values‐based attributes such as compassion and empathy. However, relational aspects of care were noted less frequently. Comments related to communication skills and values‐based care were less diverse than those relating to technical tasks and the language used was perfunctory.

**Conclusion:**

Adult nursing assessments emphasise technical, task‐focused competencies, with less attention given to relational skills like compassion and communication. To address this gap, nursing education must better integrate relational and technical competencies, enhancing patient care and supporting nurses' mental health and wellbeing for a more holistic post‐pandemic nursing practice.

**Implications for Practice:**

Results suggest an increased focus on the development of relational skills is needed for students pursuing adult nursing.

**Patient or Public Contribution:**

No patient or public contribution.

## 1 Introduction

Student nurses are expected to develop various clinical competencies so they can understand how to respond effectively to diverse health needs, unexpected events and deteriorating patients (Gellerstedt et al. 2019). This is critical as a highly skilled, diverse healthcare workforce is needed to address current epidemiological challenges (World Health Organization [WHO] 2020). Moreover, the health and social care needs of the global population are rapidly evolving (McKee et al. 2021; Sleeman et al. 2019). People are living longer, and more individuals are managing multiple coexisting complex health conditions (Chowdhury et al. 2023; Kivimäki and Frank 2023). However, the COVID‐19 pandemic disrupted healthcare systems and nurse education, creating significant challenges (Martin, Kaminski‐Ozturk, Smiley, et al. 2023; Haslam 2021). Placement opportunities for student nurses were significantly reduced due to lockdowns and social distancing rules, which limited their ability to gain hands‐on experience in clinical settings (Orazietti et al. 2023). Throughout this period, the challenges faced by student nurses were dynamic and constantly shifting. For example, fear of infection was particularly acute during the first wave, prior to vaccine availability, and perceptions of risk evolved as the pandemic progressed (Henshall et al. 2023).

In response to workforce pressures, some countries invited final‐year nursing students to take on paid roles to support healthcare services and address challenges in practice (Nursing and Midwifery Council [NMC] 2020). Placement opportunities are vital for student nurses, as they enable the application of theoretical knowledge in clinical settings, promote additional learning, and facilitate the development of competencies required by the NMC (Brett et al. 2024). The pandemic disrupted these experiences, restricting opportunities to online in some situations to practice essential skills necessary for safe patient care. This included learning to perform clinical procedures and effectively communicate with patients and their families, both of which are fundamental to the nursing role (Wittenberg et al. 2021).

Since the return of in‐person nurse education, student nurses are entering a workforce that has been profoundly altered by the COVID‐19 pandemic (Fleisher et al. 2022). For instance, the high workloads and increased stress caused by the pandemic have exacerbated pre‐existing levels of burnout among nurses (Martin, Kaminski‐Ozturk, O'Hara, et al. 2023). Unsurprisingly, there has been an observed rise in mental health conditions (Murray et al. 2020) and suicide rates among nurses (Zohn and Hovis 2024). Additionally, healthcare systems worldwide are grappling with the financial constraints of the post‐COVID era (Melnick and Maerki 2023). As a result, student nurses must navigate clinical practice placements in increasingly uncertain and resource‐limited environments. This makes the task of educating student nurses to address universal health needs holistically and equitably more complex than ever (Langenberg et al. 2023; Baker et al. 2021).

## 2 Background

In the United Kingdom, there are various routes to becoming a registered nurse; programmes are offered on a 3‐year undergraduate basis ‐ there is also a 2‐year pre‐registration degree. Students who already hold an undergraduate degree can train at postgraduate level by following a Masters programme. All courses are based on a 50/50 split between theory, followed by practice‐based learning. Student nurses are expected to achieve a foundational level of knowledge before becoming registered professionals (NMC 2018a). This includes addressing the needs of people with physical and mental health conditions (WHO 2020; NMC 2018a). Therefore, new registrants need to demonstrate proficiency in planning, assessing, and managing best practices (Loveday 2019), including theoretical and practical nursing knowledge, as well as developing an understanding of other professions (Aldriwesh et al. 2022). Research has thus far identified some of the challenges faced by student nurses and nurse educators during the COVID‐19 pandemic. For example, these include adjusting to online learning (Dziurka et al. 2022; Kaveh et al. 2022) and fear of becoming infected by COVID‐19 (Yazici and Ökten 2022).

The role of the practice assessor is a crucial component in the supervision and assessment of nursing and midwifery students. Practice assessors evaluate a student's overall performance during their practical learning experience, considering whether they have achieved the necessary proficiencies and met the expected programme outcomes. Additionally, they observe whether students consistently demonstrate the essential values associated with their chosen profession, providing a comprehensive assessment of readiness for professional practice. This role works in conjunction with practice supervisors and academic assessors as part of a collaborative ‘triple assessment’ model (for additional information, see NMC 2018b).

Given the challenging nature of healthcare environments post‐COVID (Melnick and Maerki 2023) and the expectation that all nursing students will develop clinical skills related to managing both physical and mental health (NMC 2018a), it is essential to gain insight into what is now being prioritised by nurse practice assessors. Such insights may help clarify what is being considered when assessing the future nursing workforce. However, to the authors' knowledge, there is a lack of empirical work exploring what is currently being reflected in the assessment of student nurses following the COVID‐19 pandemic (Painter and Bond 2023). Hence, the focus of the current study was to explore the priorities of registered nurse practice assessors during their assessment of students pursuing adult nursing by addressing the following research question: What skill(s) are being prioritised in the practice assessment of students pursuing adult nursing?

## 3 The Study

### 3.1 Aim

To explore comments made by registered nurse practice assessors during the clinical assessment of students pursuing adult nursing.

## 4 Methods

### 4.1 Data Collection and Analysis

Data were collected from the online practice assessment documentation of second year undergraduate students currently pursuing adult nursing and working toward a qualification in the United Kingdom at one University in the north of England. The current study used the online practice assessment document ‘MYEPAD’, which stands for ‘Midlands, Yorkshire and East Practice Assessment Document’. This was produced collaboratively between 28 universities and was developed to ensure that student nurses are prepared to successfully meet the Future Nurse: Standards of Proficiency for Registered Nurses (Nursing and Midwifery Council, NMC 2018a) at the point of registration. The MYEPAD details the level of performance that students are required to demonstrate at the end of each year of study (see South Yorkshire Primary Care Workforce and Training Hub 2018).

Sections of the online practice assessment documentation are structured to allow practice assessors to comment on students' ‘knowledge, skills, attitudes and values’. Comments were collected from a total of 27 individual practice assessors from one National Health Service (NHS) organisation. Each practice assessor made 3 comments per student (81 pieces of qualitative data) around values, knowledge and skills development. Content analysis was chosen as this allowed the team to condense large amounts of textual data into manageable categories based on explicit coding rules, whilst maintaining the depth and richness of the data (Elo et al. 2014). This offers a unique approach compared to other qualitative methods, as it allows researchers to explore both qualitative themes as well as the identification of quantitative trends/patterns in the data (Roller 2019).

Data collection and analysis were conducted simultaneously, following the phases for qualitative content analysis described by Elo and Kyngäs (2008). This proceeded as follows: data were extracted from sections of students' online practice assessment documentation. Open comments were extracted and transferred onto a Microsoft Word document. At this point, any identifying information was immediately replaced with generic terms that is, [student name] and/or [assessor/organisation name]. This activity formed the initial phase of the content analysis, whereby one researcher (CB) began analysing the data through a process of immersion (Kleinheksel et al. 2020) while transferring data to a coding sheet for later use by both researchers.

Open coding then took place (separately) by both researchers, who made notes independently within the margins of the coding sheet. Smaller content categories were identified inductively by each researcher through the identification of words or sections of text (Armat et al. 2018; Elo and Kyngäs 2008). The researchers met on two separate occasions, across a period of 1 month, to discuss all the identified content. Sub‐categories and generic categories were checked and discussed according to the aims of the study. Two main themes were identified.

Saturation in data collection was reached as the analysis progressed. As coding continued, the researchers observed that additional comments from assessors became increasingly repetitive, with no new themes being identified (Saunders et al. 2018). This indicated that further data collection would likely yield similar insights. Therefore, it was concluded that saturation had been achieved, confirming that the sample size was sufficient to address the research aim. Saturation was specifically identified when no new content or insights emerged from the data, and the themes identified were consistently supported across the dataset. The COREQ checklist was used to guide reporting (Tong et al. 2007).

### 4.2 Ethical Considerations

Ethical approval was received from the Sheffield Hallam University Ethics Committee (ID: ER53538781). Prior to collecting the data, the research team confirmed ownership of the data (host university) and, as a courtesy, data subjects were made aware of the study prior to any extraction of raw data. To ensure confidentiality and protect the identities of individuals, pseudonyms were used for all students and practice assessors in the data analysis and reporting stages of the study.

### 4.3 Trustworthiness

The authors view themselves as active participants (Bryman 2016; Chavez 2008), and the insider position is seen as a strength of the research; beneficial to the research process (Patton 2001). Their combined tacit knowledge and experience as specialist adult and mental health nurse academics are highly relevant for generating meaningful and insightful inferences during content analyses (Holmes 2020; Elo et al. 2014). Nonetheless, the authors were continuously mindful of their positionality and potential biases throughout the study's lifecycle.

To enhance the trustworthiness of this research, Lincoln and Guba's (1985) criteria for qualitative inquiry were explicitly addressed, alongside appraisal principles for rigour in qualitative studies (Moorley and Cathala 2019). Credibility was established through reflexive discussions, during which the authors critically challenged one another's positionality and subjectivity. These discussions ensured that interpretations remained grounded in the research aims and the data‐derived content (Olmos‐Vega et al. 2023). Transferability was supported by providing a clear and detailed description of the context, methodology and findings, enabling others to assess the applicability of the study's outcomes to their own settings. Dependability was upheld through a consistent and transparent approach to data analysis, with themes independently reviewed (second author) to ensure coherence and alignment with the study's objectives. Finally, confirmability was achieved by maintaining a reflexive stance, where decisions and interpretations were continuously examined to minimise researcher bias. This comprehensive approach underscores the authors' commitment to conducting a rigorous and trustworthy study, ensuring that the findings are both meaningful and credible.

## 5 Results

The analysis identified two core themes: *task‐focused competencies* and *relational/transformative aspects of care*. Within these, subthemes provided insight into the specific competencies and attributes highlighted in the data. Task‐focused competencies encompassed practical and technical nursing skills, while relational/transformative aspects of care emphasised values‐based attributes such as compassion and empathy.

### 5.1 Theme 1: Task‐Focussed Competencies

The data revealed that a larger proportion of the content fell under the task‐focused competencies theme, as shown in Table 1. This theme encompasses a broad range of competencies, from essential skills relevant to all fields of nursing, such as aseptic technique and venepuncture, to more specialised adult nursing skills, such as bladder scanning and fracture assessments. These skills were often detailed in the data, illustrating the emphasis placed on the practical, hands‐on aspects of nursing care. The focus on task‐focused competencies underlines the importance of technical skills in the student's development and the assessors' prioritisation of these competencies in the feedback provided.

**TABLE 1:** Task‐focused competencies and subthemes.

Core theme	Task‐focussed competencies	
Subthemes	Relevant to all fields of nursing	Specific to adult/general nursing
Elements of each subtheme	ECGs, physical observations (NEWS score) Medication, record keeping Learning about different conditions Aseptic technique/wound assessment Venepuncture, IV fluids, catheterisation End of life care, CPR, GCS	Bladder scanning/cystoscopy Fracture assessments Surgical care, complex/chronic wound management ENT, haematology emergencies Individualised care

#### 5.1.1 Subtheme 1: Relevant to All Fields of Nursing

Practice assessors frequently commented on a wide range of task‐focused competencies aligned with the NMC standards of proficiency for nursing, which are relevant to all fields of nursing (NMC 2018a). These included essential clinical skills such as aseptic technique, venepuncture, intravenous (IV) line management and catheterisation.

Able to recognise sepsis, escalates news scores appropriately, catheterisation using aseptic technique, starts on fluid balance charts, sends off urine for cultures. (Assessor A, Student Sophie)

[student name] has actively taken part in many clinical skills whilst she has been on placement, including the use of aseptic technique, catheterisation, administration of NG medication and wound dressings. [student name] has also practiced her basic nursing care skills such as skin assessments, participating in moving and handling and assessment of nutritional needs. (Assessor B, Student Gillian)

Uses her own initiative to commence with assessments such as Glasgow Coma Scale, blood glucose monitoring and lying and standing blood pressure. (Assessor C, Student Emily)

The extracts indicate that practice assessors prioritised the student's competence in core and specialised clinical skills, particularly those essential to patient safety and effective care. The emphasis was placed on task‐focused competencies, especially technically complex procedures such as catheterisation, aseptic technique and managing patient assessments. These skills reflect the assessors' focus on ensuring that the student can perform essential nursing tasks with accuracy and initiative.

Assessors also highlighted the student's proactive approach to care, as seen in their independent use of clinical assessments like the Glasgow Coma Scale and blood glucose monitoring. The frequent mention of these competencies suggests that assessors valued not only technical proficiency but also the ability to take initiative and apply theoretical knowledge to practice.

#### 5.1.2 Subtheme 2: Specific to Adult/General Nursing

The focus on both routine and specialist skills such as bladder scanning and fracture assessment further reinforces the importance placed on practical, hands‐on expertise in the context of adult nursing. Overall, the assessors were prioritising technical competence and initiative, which are central to the theme of *task‐focused competencies*.

[student name] has been developing basic urology nursing skills‐ bladder scanning, removing catheters, setting up for flexible cystoscopies. (Assessor D, Student Leanne)

Did full nurse assessment for patient with ankle fracture, measured for plaster of Paris. Assisted myself to apply pot explaining reasoning for why we use back slabs in ED (to allow for limb swelling). (Assessor E, Student Liam)

She has worked well in the plastic dressing clinic participating and helping with various dressing for small minor wounds, to large complex wounds that require negative pressure vacs and picco dressing. (Assessor F, Student Anna)

The extracts further demonstrate the assessors' prioritisation of task‐focused competencies, extending from essential skills to more specialist clinical practices. In addition to the basic competencies already highlighted, the assessors placed importance on the student's development in specialised areas of adult nursing, such as bladder scanning, catheter removal and setting up for flexible cystoscopies. These advanced skills show that the assessors valued the student's growing technical proficiency in more complex, context‐specific interventions, reflecting their emphasis on competence in specialist clinical areas.

The focus on technical skills extended beyond urology, with the student also gaining experience in general nursing procedures, such as performing full nursing assessments for a patient with an ankle fracture. Assisting in applying plaster casts and explaining clinical reasoning, such as using back slabs in the emergency department, highlighted the assessors' recognition of the student's ability to manage both routine and more complex tasks. This aligns with the earlier emphasis on the value of technical ability and an understanding of the clinical rationale behind nursing procedures. Additionally, the student's participation in the plastic dressing clinic further illustrates their involvement in managing complex wounds, including those requiring advanced interventions like negative pressure vacs and Picco dressings. Together, these comments underscore assessors' prioritisation of a broad range of task‐focused competencies, ranging from basic nursing procedures to more specialised clinical interventions, all of which contribute to the student's overall development in adult nursing practice.

### 5.2 Theme 2: Relational/Transformative Competencies

The data indicated that relational/transformative competencies were less frequently detailed than task‐focused competencies; feedback consistently recognised these competencies, showing that assessors valued not only the student's technical skills but also their ability to engage with patients and colleagues in a compassionate, respectful manner, demonstrating the essential relational skills needed in nursing practice. While emphasis in this theme was placed on personal attributes such as communication, compassion and professionalism, as shown in Table 2, these values‐based attributes were highlighted, though comments tended to be more succinct and less elaborated upon.

**TABLE 2:** Relational/transformative aspects of care and subthemes.

Core theme	Relational/transformative aspects of care		
Subthemes	Personal attributes and characteristics	Values	Work ethic
Elements of each subtheme	Communication Calm/polite Good/positive attitude	Compassion Dignity/respect Professional/NMC code/policy	Team player, punctual and presentable Keen to learn, proactive

#### 5.2.1 Subtheme 1: Personal Attributes and Characteristics

The data revealed that communication skills were frequently commented on, though assessors' feedback was generally brief and lacked elaboration on how or why communication was used in practice. While many assessors highlighted the student's ability to communicate effectively, the feedback did not go into detail about the specific situations or contexts in which communication played a critical role in patient care. For instance, comments such as ‘Good communication skills’ or ‘Communicates well with patients’ were common, but they did not provide insight into how these skills were applied to improve patient outcomes. This suggests that while communication is recognised as important, its role in enhancing the therapeutic relationship or contributing to patient care wasn't fully explored in the feedback.

Good communication skills. (Assessor G, Student Olivia)

[student name] has also communicated well with the MDT within the department. (Assessor H, Student Nathan)

Communicates well with patients. (Assessor A, Student Maya)

She has communicated well, and appropriately with both staff and patients. (Assessor I, Student Collette)

However, when communication was noted in more specific contexts, such as in the use of SBAR handovers or in escalating patient concerns, the feedback was more detailed. The emphasis was on the student's ability to relay important information to the multidisciplinary team (MDT) and escalate concerns when necessary, such as reporting high NEWS scores to doctors. These more specific comments suggest that assessors valued communication in terms of its functional role in ensuring patient safety and continuity of care, rather than as a relational skill or as part of building rapport with patients.

Her communication skills are already excellent, she has been able to put these skills into practice with caring for patients and handing over to the ward staff. (Assessor G, Student Ella)

Her communication skills are excellent, she is able to undertake an SBAR handover with ease and confidently escalates any concerns to the appropriate person for example, informing Dr's of high NEWS scores. (Assessor J, Student Chloe)

#### 5.2.2 Subtheme 2: Values

Alongside communication, the student's values, including compassion, dignity and respect, were frequently mentioned by assessors. These values are often described in succinct terms, without further qualification or examples. For instance, comments reflected the student's adherence to core nursing values, but the feedback lacked depth in explaining how these values were demonstrated in practice or their impact on patient care, as these first two extracts illustrate:

Very professional‐ is compassionate, treats patient with respect. (Assessor K, Student Ava)

She shows compassion and dignity to patients she cares for. (Assessor L, Student Mia)

[student name] has provided individualised care to her patients she has treated them with respect and maintained privacy and dignity, she has offered choice and gained consent when needed. (Assessor M, Student Nancy)

The final quote sees the use of the term ‘individualised care’, which appeared only once in the data. This might suggest that while assessors acknowledged the student's values‐based care, they did not extensively elaborate on how these values were applied in diverse clinical situations. Similarly, the use of the term ‘holistic’ was rare, reinforcing the idea that while these relational competencies were recognised, they were not deeply explored or elaborated upon in the feedback.

Another consistent element of this subtheme was adherence to professional standards, including the NMC's Code of Conduct. Assessors noted the student's alignment with the NMC code and local Trust policies, which highlight the importance of professional conduct in nursing practice. The quotes below emphasise the assessor's consideration of the student's professionalism and ethical approach to patient care. This adherence to professional standards appears to be a central aspect of the assessors' feedback, suggesting that the student's relational competencies were strongly aligned with the ethical and legal frameworks that govern nursing practice.

(student name) has displayed high professional values and standards throughout this placement. She works in line with the NMC code and local policies. She always demonstrates compassion and builds a good rapport with patients. (Assessor N, Student Charlotte)

(student name) understands the importance of adhering to the Trust policies and procedures and also the NMC Code. She also understands the importance of maintaining patient confidentiality. She upholds these attitudes during her placement. (Assessor B, Student Isla)

She was always on time, looking professional and her behaviours and attitudes were in line with [organisation name] requirements. (Assessor O, Student Lily)

#### 5.2.3 Subtheme 3: Work Ethic

Finally, the subtheme of work ethic was reflected in assessors' comments; frequently commenting on the student's attitude toward learning. Words such as ‘punctual’, ‘organised’ and ‘motivated’ were commonly used to describe the student's approach to their placement.

[student name] is consistently punctual and organised, approaches each task with a positive, motivated attitude, demonstrating a clear commitment to learning and improving. (Assessor P, Student Amelia)

[Student name] is proactive in seeking learning opportunities and approaches each task with enthusiasm, ensuring she contributes effectively to the team. (Assessor Q, Student Hayley)

is always organised and consistently punctual. Always wears correct uniform and is committed to learning. (Assessor C, Student Dawn)

is proactive in seeking learning opportunities and approaches each task with enthusiasm, ensuring she contributes effectively to the team. (Assessor R, Student Fiona)

The comments suggest that the assessors valued the student's proactive and professional attitude, indicating that work ethic is seen as a key component of relational/transformative competencies. The focus on work ethic further reflects the importance of professionalism, reliability, and a commitment to essential qualities in nursing practice.

## 6 Discussion

This study aimed to explore the comments made by adult/general nurse practice assessors within the practice assessment documentation of students pursuing adult nursing. The findings highlight a clear emphasis on the acquisition of task‐focused skills, particularly those related to physical health and technically complex procedures. These results indicate that adult nursing assessments prioritise physical health skills, with more data falling within the task‐focused competencies subtheme (Table 1). This trend aligns with previous research, such as Lamph et al. (2023), which found that adult nursing emphasises transactional and physical health competencies over relational aspects of care. Similarly, an earlier work (Painter and Bond 2023) observed that complex physical health skills were less evident in the assessment of students pursuing mental health nursing. Additionally, this trend reflects broader shifts in healthcare delivery during the COVID‐19 pandemic, which led to a greater focus on technical skills due to increased patient acuity and reduced opportunities for relational skill development (Henshall et al. 2023).

The findings also show that values‐based aspects of care, such as compassion, were briefly mentioned in assessments of adult nursing students, but these comments were often generic and lacked detail. For example, statements like ‘Good communication skills’ or ‘is compassionate’ were common, but rarely included elaboration on how these skills were demonstrated. This contrasts with research on mental health nursing, where assessments of compassion and communication skills tend to be more detailed (Painter and Bond 2023). Relational competencies such as communication and compassion are widely acknowledged as essential to effective healthcare delivery (Sinclair et al. 2016). However, the current findings suggest these skills are less prioritised in adult nursing assessments, potentially reflecting differing expectations between specialisms.

Several factors may explain this divergence. Adult nursing has traditionally prioritised complex physical health skills, which may account for their prominence in assessments. In contrast, technical physical skills are a relatively recent addition to mental health nursing curricula (Dickens et al. 2019). The COVID‐19 pandemic likely further shifted focus toward physical health, with relational skills deprioritised due to restricted practice opportunities and the increased urgency of managing patient acuity (Davey et al. 2022; Wittenberg et al. 2021). Broader systemic challenges, including workforce attrition, burnout and the rising complexity of health conditions, may also contribute to the reduced emphasis on relational competencies (McKee et al. 2021; Martin, Kaminski‐Ozturk, O'Hara, et al. 2023; Martin, Kaminski‐Ozturk, Smiley, et al. 2023). Furthermore, articulating and assessing relational skills such as compassion can be inherently challenging. Even within mental health nursing, where humanistic care is a central focus, defining and demonstrating compassion across different contexts remain complex (Marshman et al. 2021).

A disparity in how competencies are assessed may further explain the limited focus on relational skills. The NMC's (2018a) standards for registered nurses emphasise both task‐focused and relational competencies, including technical proficiency, communication skills and compassionate care. However, the operationalisation of these standards in assessment tools may disproportionately favour technical skills, as they are often easier to measure. This imbalance could lead to relational competencies being underrepresented in assessment documentation, despite their importance to holistic, patient‐centred care.

Regardless of these challenges, all student nurses must develop physical and mental healthcare competencies, as outlined by global health frameworks (WHO 2020; Moyo et al. 2022; NMC 2018a). However, the current study suggests that relational skills, which are integral to person‐centred care, are underrepresented in adult nursing assessments. For example, the concept of holism was mentioned only once in the data, and mental health assessments were notably absent. This gap is significant, given that relational skills such as communication, interpersonal skills and active listening are associated with improved perceptions of care quality (Malenfant et al. 2022) and better recovery outcomes for patients with mental health conditions (Bond et al. 2024). These findings are especially pertinent considering the global rise in mental health conditions following the pandemic, including increased suicide rates among the general population and healthcare workers (Murray et al. 2020; Zohn and Hovis 2024). Addressing mental health in healthcare settings and prioritising the wellbeing of nurses alongside that of patients is now more critical than ever.

Although the findings of this study emphasise task‐focused competencies, they also underscore the need for a more balanced approach in nursing education. Brett et al. (2024) argue that while technical skills are vital for ensuring patient safety and quality care, relational competencies such as communication, empathy and compassion are equally crucial for delivering holistic care. The current study, however, highlights a potential gap in the assessment of relational skills among adult nursing students. Bridging this gap will require educational frameworks that integrate relational and technical competencies, ultimately preparing nurses to meet the complex and evolving demands of healthcare.

### 6.1 Strengths and Limitations

This study offers several strengths alongside opportunities for further exploration. The use of online assessment documentation with a tick‐box structure provided a systematic way to analyse how assessors prioritise competencies in practice settings. While this approach may simplify complex relational skills, it also enabled the management of large, nuanced datasets; a recognised strength of content analysis for capturing patterns in multidimensional phenomena like nursing work (Elo and Kyngäs 2008). Through the employment of an inductive approach to category identification, validated through independent review by a third researcher (Armat et al. 2018; Elo et al. 2014), the study ensured methodological transparency and rigour (Moorley and Cathala 2019). Furthermore, the researchers' positionality as specialist nurse academics enhanced the interpretive depth of the analysis, as their tacit knowledge allowed for meaningful inferences about assessment priorities (Holmes 2020; Elo et al. 2014).

The sample size of 81 qualitative data points from 27 assessors aligns with established guidelines for content analysis (Bengtsson 2016), offering credible insights into local assessment practices. While the single‐site design limits generalisability to national or global contexts, it provides a focused lens for understanding institutional priorities, with clear potential to inform improvements in assessment frameworks within similar settings. The findings highlight how task‐focused competencies may dominate documentation, raising critical questions about how relational skills, such as compassion, are observed and valued in practice.

To build on these insights, mixed‐methods approaches could deepen the understanding of assessors' perspectives. For example, integrating ethnography or interviews with practice assessors would allow exploration of implicit biases, contextual influences, and the ‘how’ of relational skill enactment, topics less visible in written documentation. Comparative studies across institutions could further clarify whether the observed prioritisation of task‐based competencies reflects broader trends or local practices. Additionally, research into how nursing curricula translate into real‐world assessment behaviours could bridge gaps between educational aims and workplace priorities.

## 7 Conclusion

In conclusion, while adult nursing assessments prioritise the acquisition of technical, task‐focused competencies, there is a notable gap in the emphasis on relational skills such as compassion and communication. Given the increasing importance of mental health in global health agendas and the impact of relational care on both patients and healthcare providers, it is essential that future nursing education frameworks better integrate relational competencies alongside technical skills. Addressing this imbalance will not only enhance the quality of patient care but also support the mental health and wellbeing of nurses themselves, ensuring a more holistic approach to nursing practice in the post‐pandemic world.

## Ethics Statement

Sheffield Hallam University Research Ethics Committee approved this study on 15 May 2023 (ID: ER53538781).

## Conflicts of Interest

The authors declare no conflicts of interest.

## Data Availability

Research data are not shared.
